# Renal replacement therapy in acute kidney injury from a Chinese cross-sectional study: patient, clinical, socioeconomic and health service predictors of treatment

**DOI:** 10.1186/s12882-017-0567-9

**Published:** 2017-05-04

**Authors:** Fang Wang, Daqing Hong, Yafang Wang, Yunlin Feng, Li Wang, Li Yang, ISN AKF 0 by 25 China Consortium, Li Yang, G Xing, L Wang, Y Wu, Suhua Li, G Xu, Q He, J Chen, M Chen, X Liu, Z Zhu, Lin Yang, X. Lian, F. Ding, Y. Li, Huamin Wang, Jianqin Wang, R. Wang, C. Mei, Jixian Xu, R. Li, J. Cao, L. Zhang, Yan Wang, Jinhua Xu, B. Bao, Bicheng Liu, H. Chen, Shaomei Li, Y. Zha, Q. Luo, D. Chen, Y. Shen, Y. Liao, Z. Zhang, X. Wang, K. Zhang, L. Liu, P. Mao, C. Guo, J. Li, Z. Wang, S. Bai, S. Shi

**Affiliations:** 1 Division of Nephrology, Sichuan Academy of Medical Sciences & Sichuan Provincial People’s Hospital, Chengdu, 610072 China; 2 Renal Division, Department of Medicine, Peking University First Hospital, Beijing, 100034 People’s Republic of China; 3 Key Laboratory of Renal Disease, Ministry of Health of China, Beijing, 100034 People’s Republic of China

**Keywords:** Acute kidney injury, Hemodialysis, Peritoneal dialysis, Renal replacement therapy, Developing countries, China

## Abstract

**Background:**

Renal replacement therapy (RRT) is important to support critically ill patients with acute kidney injury (AKI). This study, a part of a nation-wide survey for AKI conducted by the ISN AKF 0 by 25 China Consortium, aims to study the current RRT practical situation and problems in China.

**Methods:**

The current study is a part of a nation-wide survey for AKI conducted by ISN AKF 0 by 25 China Consortium. The survey included 44 sites all over the country, including 22 academic hospitals in big cities and 22 local hospitals in smaller cities or rural areas. Of the 44 sites, all have access to PD and IHD, 93.5% are capable to perform CRRT. Of total 7604 AKI cases, 896 cases (11.8%) had indications for RRT and were included in the current abstract.

**Results:**

of the 896 patients that had indications for RRT, only 59.3% received RRT. Patients who were older, male, from lower income areas, in local hospitals, or with severe comorbidities, were less likely to receive RRT. RRT treatment was associated with lower mortality (OR = 0.58, 95%CI 0.38–0.89). The RRT modalities were continuous renal replacement therapy (CRRT) in 53.9%, intermittent hemodialysis (IHD) in 38.0%, CRRT complemented by IHD in 6.2%, CRRT complemented by peritoneal dialysis (PD) in 0.8% and PD in 1.1%. Of the subgroup of patients receiving RRT who did not have an indication for modality of CRRT, 36.8% in fact received CRRT, and their medical cost and mortality rate was higher (7944[4248, 16,055] vs. 5100[2948, 9396] US dollars, *p* < 0.001 and 10.6% vs. 4.4%, *p* = 0.047, respectively) compared with those treated with other RRT modalities).

**Conclusions:**

Extrapolated to the whole of China our results indicate that an estimated 139,000 patients with an indication of RRT are under treated without RRT over a year. Non-clinical factors influence RRT prescription for severe AKI patients. CRRT may be over-utilized in the treatment of severe AKI and the use of PD is extremely rare. These findings have implications for the effective application of medical resources in the treatment of severe AKI.

## Background

Acute kidney injury (AKI), a “Silent killer” [1], is getting more and more attention because of its increasing incidence and adverse impact on patients’ outcome and health cost burden [2–7]. However, AKI are still under-recognized and/or under-treated, especially in the developing countries, due to low awareness and low medical resources [8].

Renal replacement therapy (RRT), as a supportive management, remains the main treatment strategy for severe AKI patients. Although debates on the optimal timing to initiate RRT and the optimal choice of RRT modalities still continue, knowledge must be known about the current situation lagging behind the strategy available nowadays, and efforts need to be made to improve this situation which is as important as optimizing the management which is relied on future research.

In order to improve the diagnosis and treatment of AKI globally, International Society of Nephrology (ISN) carried out a global target of 0 by 25-zero death of patients with untreated acute kidney failure by 2025. As part of this project, we carried out the largest nation-wide survey for AKI in China. In order to reveal the current status and further improve the situation of RRT practice for the AKI patients in China, we performed a sub-analysis on the data collected from the survey, including 2,223,230 hospitalized adult patients from 22 provinces, municipalities or autonomous regions in Mainland China [9].

## Methods

### Participants

**Fig. 1:**
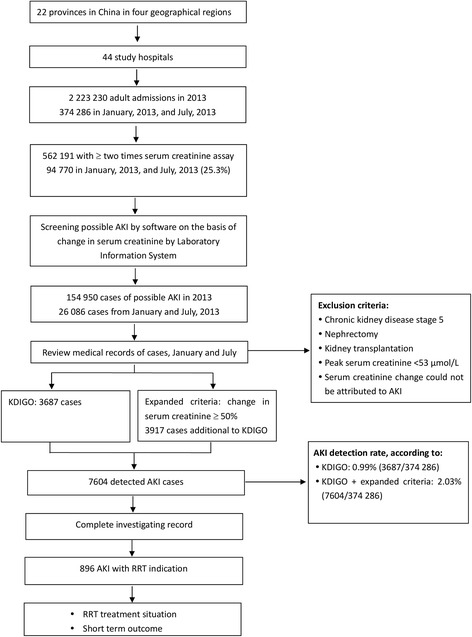
Study profile. Abbreviations: AKI, acute kidney injury; RRT, renal replacement therapy. Notes: This figure was modified with permission from the original report (9)

Patients were derived from a cross-sectional survey from 22 Chinese provinces, municipalities or autonomous regions, which covered 82% of the country’s population and four geographic regions of China (North, Northwest, Southwest, and Southeast) [9]. 2,223,230 adult patients (> = 18 years) were admitted in 44 study hospitals during 2013, among which 26,086 cases were reviewed with medical records. AKI was diagnosed in 7064 cases, of which 896 patients with renal replacement therapy indications were finally enrolled in this sub-analysis (Study profile, Fig. 1). All the study hospitals that were enrolled in the nation-wide survey were general hospitals with nephrology specialty and facilities for hemodialysis (HD), and peritoneal dialysis (PD). Continuous renal replacement therapy (CRRT) was available in 93.5% of the hospitals. The study protocol and waiver of patient informed consent was approved by the ethic committees of Peking University First Hospital and the enrolled study hospitals. Required data for this study was obtained in a de-identified and anonymized form.

### Diagnosis criteria

Three steps were carried out to accomplish this national survey. Firstly, serum creatinine (SCr) was reported by the Laboratory Information System and changes in serum creatinine (△SCr) were evaluated to screen AKI. Secondly, hospital records of the suspected patients were reviewed by trained nephrologists/renal fellows to confirm the diagnosis of AKI. Thirdly, relevant records (such as demographic information, comorbidities, clinical departments, in-hospital costs and outcomes, etc.) were completed for the patients confirmed with AKI.

AKI was diagnosed according to either 2012 KDIGO AKI definition [10] (criteria 1) or an expanded criterion as defined by authors: an increase or decrease in SCr by 50% during hospital stay (criteria 2). Patients with baseline CKD stage 5, previous nephrectomy, kidney transplantation, peak SCr < 0.6 mg/dl or changes of SCr not attributed to AKI (such as SCr decrease after amputation, etc.) were excluded. Severe comorbidity was defined as having either of the following clinical situations: multiple organ dysfunction syndrome (MODS), disseminated intravascular coagulation, sepsis, advanced stage of malignancies, acute respiratory distress syndrome, shock or mechanical ventilation.

The indications for RRT included1) volume overload unresponsive to diuretic therapy; or 2) severe hyperkalemia (≥6 mmol/L) or metabolic acidosis (pH ≤ 7.3); or 3) BUN ≥ 60 mmol/L or 4) overt uremic manifestations such as pericarditis and encephalopathy. In additions to the above traditional indications for CRRT, if patients had at least one of the following commobidities: rhabdomyolysis, sepsis, MODS, respiratory failure requiring mechanical ventilation, shock, CRRT were also indicated as RRT could potentially benefits these patients.

Renal recovery at discharge was classified as: 1) full recovery, defined as SCr fell below threshold or to the baseline; 2) partial recovery, defined as SCr decreased by ≧25% from peak level but remained above the threshold or baseline; 3) failure to recover, defined as dialysis dependent or SCr decreased by < 25% from peak level. In-hospital mortality was defined as recorded death in medical records. Details in the study design and working process was present in the previous publication [9].

### Statistical analysis

Continuous data were presented as means with SDs or medians (IQR). Categorical variables were presented as proportions. Continuous data were compared between two groups using t-test or non-parametric Wilcoxon rank sum test for severely skewed data. Chi-square test was used to compare categorical variables between two groups. In this study, demographic characteristics, comorbidities, in-hospital covariates (such as admission department, renal consultancy, etc.), degree of disease, renal recovery, in-hospital mortality and costs were compared between RRT treatment group and no-RRT treatment group. Multiple logistic regression was applied to study the factors associated with no-RRT treatment using variables including age, gender, gross domestic product (GDP) per head, academic hospital, renal referral, AKP peak stage, hospital-acquired AKI, non-oliguria and severe comorbidities. The relationship between RRT and in-hospital mortality was analyzed with multiple logistic regression adjusting for age, gender, GDP per head, academic hospital, renal referral, AKI peak stage, hospital-acquired AKI, cardiovascular disease (CVD), diabetes mellitus (DM), non-oliguria and severe comorbidities. RRT modalities were divided into CRRT, CRRT + IHD, CRRT + PD, IHD and PD, and Chi-square was utilized to compare the difference of RRT modalities among departments including ICU, renal and other departments. Age, gender, AKI peak stage, severe comorbidities, costs, renal referral, in-hospital and short-term mortality and cost were compared between CRRT and other-RRT-treated groups in patients without CRRT indications. A two tailed *P* value <0.05 was regarded as indicative of statistical significance. All analyses were performed using SAS software (version 9.1, SAS Institute, Cary, NC, USA).

## Results

### Characteristics of participants with RRT indications (Table 1)

Of total 7604 AKI cases, 896 cases (11.8%) had indications for RRT and were included in the current study. The mean age was 59.7 ± 18.2 years. 65.5% were male patients baseline chronic kidney disease (CKD) was in 34.2% of patients. 41.5% of patients had hypertension while 20.0%haddiabetes mellitus Most of the patients were hospitalized in academic hospitals (81.7%). The percentage of the patients who were admitted to intensive care unit (ICU), renal, surgical and other medical departments were 42.2%, 17.9%, 11.6% and 28.3% respectively. Most of the patients (72.1%) had AKI stage 3, and the rest were at AKI stage 2 (14.5%) or stage 1 (13.4%), with 516 (57.6%) patients having severe comorbidities. Only 57.8% of the patients (*n* = 518) had renal referral. There were 216 patients (216/859, 25.1%) died during their hospital stay (37 patients without identifiable outcome) and 200 (200/859, 23.3%) cases were recorded as ‘treatment withdrawal’. Patients in male gender (23.9% vs. 22.0%, *p* = 0.515), with malignancies (28.7% vs. 22.3%, *p* = 0.105), with severe comorbidities (28.7% vs. 16.2%, *p* < 0.001), and from lower income areas (24.5% tertile1 vs. 27.1% tertile2 vs. 17.5% tertile3, *p* = 0.020) were more likely to withdraw treatment. Among the survived patients who had identifiable record for renal recovery at discharge (*n* = 615), 126 patients (20.5%) got full renal recovery and 203 cases (33.0%) got partial recovery.

**Table 1:** Characteristics of participants with RRT indications

	Total (*n* = 896)	RRT(*n* = 531)	No-RRT(*n* = 365)	*P*
Age, years	59.7 ± 18.2	57.0 ± 18.2	63.7 ± 17.4	<0.001
Male sex	587 (65.5%)	322 (60.6%)	265 (72.6%)	<0.001
GDP per head^a^				<0.001
Tertile 1	294 (32.8%)	139 (26.2%)	155 (42.5%)	
Tertile 2	319 (35.6%)	192 (36.2%)	127 (34.8%)	
Tertile 3	283 (31.6%)	200 (37.7%)	83 (22.7%)	
HA- AKI	360 (40.2%)	184 (34.7%)	176 (48.2%)	<0.001
CKD	306 (34.2%)	185 (34.8%)	121 (33.2%)	0.600
HT	372 (41.5%)	214 (40.3%)	158 (43.3%)	0.373
DM	179 (20.0%)	106 (20.0%)	73 (20.0%)	0.989
CVD	267 (29.8%)	138 (26.0%)	129 (35.3%)	0.003
Malignancies	143 (16.0%)	72 (13.6%)	71 (19.5%)	0.018
Academic hospital	732 (81.7%)	447 (84.2%)	285 (78.1%)	0.020
AKI stage at peak				<0.001
1	120 (13.4%)	48 (9.0%)	72 (19.7%)	
2	130 (14.5%)	54 (10.2%)	76 (20.8%)	
3	646 (72.1%)	429 (80.8%)	217 (59.5%)	
Admission dept.				<0.001
ICU	378 (42.2%)	258 (48.6%)	120 (32.9%)	
Renal	160 (17.9%)	127 (23.9%)	33 (9.0%)	
Surgical	104 (11.6%)	53 (10.0%)	51 (14.0%)	
Other	254 (28.3%)	93 (17.5%)	161 (44.1%)	
Renalreferral	518 (57.8%)	362 (68.2%)	156 (42.7%)	<0.001
Cost, US dollars	7190 (3346,16431)	9491 (4284,18905)	4537 (2405,11036)	<0.001
Classification				0.048
Pre-renal	345 (38.5%)	185 (34.8%)	160 (43.8%)	
Intra-renal	437 (48.8%)	276 (52.0%)	161 (44.1%)	
Post-renal	87 (9.7%)	52 (9.8%)	35 (9.6%)	
Unclassified	27 (3.0%)	18 (3.4%)	9 (2.5%)	
Non-Oliguria	368 (48.7%)	194 (43.1%)	174 (57.0%)	<0.001
Severe comorbidity	516 (57.6%)	269 (50.7%)	247 (67.7%)	<0.001
Renal recovery (*n* = 615)				0.094
Full recovery	126 (20.5%)	71 (17.9%)	55 (25.2%)	
Partial recovery	203 (33.0%)	134 (33.8%)	69 (31.7%)	
Failed recovery	286 (46.5%)	192 (48.4%)	94 (43.1%)	
Treatment withdrawal (*n* = 859)	200 (23.3%)	99 (19.4%)	101 (28.9%)	0.001
In hospital Mortality	216 (25.1%)	94 (18.4%)	122 (35.0%)	<0.001

*Abbreviations: RRT* renal replacement therapy, *GPD* gross domestic product, *HA-AKI* hospital acquired-acute kidney injury, *CKD* chronic kidney disease, *HT* hypertension, *DM* diabetes mellitus, *CVD* cardiovascular disease, *ICU* intensive care unit

^a^GDP per head was divided into tertiles calculated from the whole survey population

### Comparison between participants with RRT or no-RRT treatment

Of the 896 patients that had indications for RRT, only 531 cases (59.3%) received RRT (Table 1). Compared with those who did not receive RRT, patients that were treated were younger (57.0 ± 18.2 vs. 63.7 ± 17.4, *p* < 0.001), less male predominant (60.6% vs. 72.6%, *p* < 0.001), and with less pre-existing cardiovascular disease (26.0% vs. 35.3%, *p* = 0.003). They were also featured as more of oliguria (56.9% vs. 43.0%, *p* < 0.001), reaching higher AKI stage at peak (80.8% vs. 59.5% at stage 3, *p* < 0.001), and having higher intra-renal AKI proportion (52.0% vs. 44.1%, *p* = 0.048), whereas with less patients having severe comorbidities (50.7% vs. 67.7%, *p* < 0.001). RRT was performed more in academic hospitals than in local hospitals (61.1% vs. 51.2%, *p* = 0.020), more in renal department (79.4%) than in ICU (68.3%), surgical (51.0%) or other medical departments (36.6%, *p* < 0.001), and more in patients whose AKI being diagnosed by the doctors in charge or via nephrology consultation (69.9% vs. 44.7%, *p* < 0.001) than those who did not have. Patients from the lower income areas received RRT less frequently than those from the high-income areas (*p* < 0.001).Thein-hospital all-cause mortality (18.4% vs. 35.0%, *p* < 0.001) and the proportion of “treatment withdrawal” (19.4% vs. 28.9%, *p* = 0.001) was lower, and the in-hospital cost was higher in the patients who received RRT as compared to those who did not (*p* < 0.001). For the patients survived at discharge, renal recovery was not significantly different between those with and without RRT treatment (*p* = 0.094).

### Factors associated with no-RRT treatment in patients with RRT indications

Multiple logistic regression analysis (Table 2) showed that factors that might be associated with no-RRT treatment were older age (OR = 1.41 per 10 years older, 95% CI 1.18–1.70, *p* < 0.001), male gender (OR = 1.57, 95% CI 1.02–2.23, *p* = 0.012), from lower income areas (OR = 1.86 tertile2, 95% CI 1.21–2.86, *p* = 0.005; OR = 3.08 tertile1, 95% CI 2.02–4.68, *p* < 0.001), hospital-acquired AKI (OR = 1.28, 95% CI 0.98–1.82, *p* = 0.172), non-oliguric AKI (OR = 1.65, 95% CI 1.19–2.30, *p* = 0.003), and severe comorbidities (OR = 1.60, 95%CI 1.12–2.28, *p* = 0.010). Patients with AKI stage 3 at peak (OR = 0.53, 95%CI 0.32–0.88, *p* = 0.013), admitted in academic hospitals (OR = 0.53, 95% CI 0.35–0.81, *p* = 0.004) or had renal referral (OR = 0.40, 95% CI 0.28–0.56, *p* < 0.001) tended to be at lower risk of no-RRT treatment.

**Table 2:** Multiple logistic regression of non-RRT treatment

Covariate	OR	95% CI
Age (per 10 years older)	1.41 (1.18–1.70)	<0.001
Male (vs. female)	1.57 (1.10–2.23)	0.012
GDP per head		
Tertile1	3.08 (2.02–4.68)	<0.001
Tertile2	1.86 (1.21–2.86)	0.005
Tertile3	Reference	
Academic hospital	0.53 (0.35–0.81)	0.004
Renal referral	0.40 (0.28–0.56)	<0.001
AKI peak stage		
Stage 1	Reference	
Stage2	1.08 (0.58–1.99)	0.816
Stage 3	0.53 (0.32–0.88)	0.013
HA-AKI	1.28 (0.90–1.82)	0.172
Non-oliguria	1.65 (1.19–2.30)	0.003
Severe comorbidities	1.60 (1.12–2.28)	0.010

*Abbreviations: RRT* renal replacement therapy, *GPD* gross domestic product, *AKI* acute kidney injury, *HA-AKI* hospital acquired-acute kidney injury

Multivariate logistic regression was adjusted for age (every 10 year increment), gender (female as reference), income (tertile 3 as reference), academic hospital (yes vs. no), renal referral (yes vs. no), AKI peak stage (Stage1 as reference), HA-AKI (yes vs. no), Non-oliguria (yes vs. no), severe comorbidities (yes vs. no)

### Risk factors associated with in-hospital mortality (Table 3)

Multiple logistic regression analysis showed that older age (OR = 1.61 per 10 years older, 95% CI 1.26–2.05, *p* < 0.001), severe comorbidities (OR = 3.85, 95% CI 2.38–6.23, *p* < 0.001), hospital acquired AKI (OR = 3.00, 95% CI 1.98–4.56, *p* < 0.001), and higher AKI stage at peak (stage 3 vs. stage 1, OR = 2.20, 95% CI 1.14–4.25, *p* = 0.019) were independent risk factors for in-hospital mortality, while RRT treatment (OR = 0.58, 95% CI 0.38–0.89, *p* = 0.013) and renal referral were protective factors against in-hospital death (OR = 0.54, 95% CI 0.36–0.82, *p* = 0.004).

**Table 3:** multiple logistic regression of in-hospital mortality

Covariate	OR(95% CI)	*P*
Age (per 10 years older)	1.61 (1.26–2.05)	<0.001
Male	1.39 (0.90–2.14)	0.139
Income		
Tertile1	0.80 (0.48–1.33)	0.390
Tertile 2	0.66 (0.40–1.09)	0.105
Academic hospital	1.13 (0.68–1.88)	0.648
Renal referral	0.54 (0.36–0.82)	0.004
AKI peak stage		
Stage2	1.97 (0.94–4.13)	0.074
Stage 3	2.20 (1.14–4.25)	0.019
HA-AKI	3.00 (1.98–4.56)	<0.001
CVD	1.53 (0.99–2.35)	0.055
DM	1.48 (0.92–2.37)	0.104
Non-oliguria	0.68 (0.45–1.02)	0.059
Severe comoridities	3.85 (2.38–6.23)	<0.001
RRT	0.58 (0.38–0.89)	0.013

Multivariate logistic regression was adjusted for age (every 10 year increment), gender (female as reference), income (tertile 3 as reference), academic hospital (yes vs. no), renal referral (yes vs. no), AKI peak stage (Stage1 as reference), HA-AKI (yes vs. no), CVD (yes vs. no), DM (yes vs. no),non-oliguria (yes vs. no), severe comorbidities (yes vs. no) and RRT (yes vs. no)

*Abbreviations: AKI* acute kidney injury, *HA-AKI* hospital acquired-acute kidney injury, *CVD* cardiovascular disease, *DM* diabetes mellitus, *RRT* renal replacement therapy

### RRT modalities

The modalities of RRT included continuous renal replacement therapy (CRRT) in 286 cases (53.9%), CRRT complemented by intermittent hemodialysis (IHD) in 33 cases (6.2%), CRRT complemented by peritoneal dialysis (PD) in 4 cases (0.8%), IHD only in 202 cases (38.0%), and PD only in 6 cases (1.1%) (Table 4). Nearly 3/4(233/323, 72.1%) of CRRT (CRRT/CRRT + IHD/CRRT + PD) was performed in the ICU. The modalities of RRT varied among different departments. There was a greater proportion of CRRT (CRRT/CRRT + IHD/CRRT + PD) in the ICU than in the renal or other departments (90.3% vs. 22.0% vs. 42.5%, *p* < 0.001), and a greater proportion of IHD in the renal department than that in the ICU or other departments (76.4% vs. 9.3% vs. 55.5%, *p* < 0.001). PD was not compared among different departments because of the low frequency of less than 5.

**Table 4:** RRT-modalities

Clinical units	All (*n* = 531)	ICU (*n* = 258)	Renal(*n* = 127)	Others (*n* = 146)	*P*
RRT modalities					<0.001*
CRRT	286 (53.9%)	221 (85.7%)	18 (14.2%)	47 (32.2%)	
CRRT + IHD	33 (6.2%)	11 (4.3%)	8 (6.3%)	14 (9.6%)	
CRRT + PD	4 (0.8%)	1 (0.4%)	2 (1.6%)	1 (0.7%)	
IHD	202 (38.0%)	24 (9.3%)	97 (76.4%)	81 (55.5%)	
PD	6 (1.1%)	1 (0.4%)	2 (1.6%)	3 (2.1%)	

*Abbreviations: RRT* renal replacement therapy, *CRRT* continuous renal replacement therapy, *IHD* intermittent hemodialysis, *PD* peritoneal dialysis, *ICU* intensive care unit

^*^CRRT + PD and PD were not included in the Chi-square analysis because of the low frequency less than 5

### RRT modalities in patients with or without CRRT indications

Among the patients who were prescribed CRRT (*n* = 323), 204 cases (63.2%) had CRRT indications that included at least one of the comorbidities such as rhabdomyolysis, sepsis, multiple organ dysfunction syndrome (MODS), respiratory failure needing mechanical ventilation, or shock. On the other hand, of the 250patients who had CRRT indications, 46 cases (18.4%) were treated with IHD, among whom 9 cases (9/46, 20%) left hospital with “treatment withdrawal” soon afterwards, indicating an influence of economic conditions on the choice of RRT treatment.

Among the 119 patients that had no CRRT indications but prescribed with CRRT, half (*n* = 60, 50.4%) were hospitalized in ICU and the others were admitted to renal (*n* = 22, 18.5%), surgical (*n* = 6, 5.0%) and other medical units (*n* = 31, 26.1%). Among the various clinical units, more patients that had no CRRT indications were treated with CRRT in the ICU (60/71, 84.5%) compared with those in renal (22/110, 20.0%), surgical (6/33, 18.2%) and other medical units (31/67, 46.3%), (*p* < 0.001).

Of the 281 patients that had no CRRT indications, renal referrals were lower in the CRRT prescriptions than the IHD prescriptions (67.2% vs. 84.6%, *p* = 0.001). There was no significant difference in the AKI peak stage or the proportion of severe comorbidities between the patients with CRRT treatment (*n* = 119) and those with other RRT treatments (*n* = 162), whereas the in-hospital mortality rate (10.6% vs. 4.4%, *p* = 0.047) and the overall medical costs (7944[4248, 16,055] vs. 5100[2948, 9396] US dollars, *p* < 0.001) were higher in the patients with CRRT treatment (Table 5).

**Table 5:** New Comparison between CRRT-treated and other-RRT-treated patients without CRRT indications

	CRRT-treated (*n* = 119)	Other-RRT-treated (*n* = 162)	*P*
Age, years	57.3 ± 18.6	54.6 ± 16.6	0.199
Male sex	68 (57.1%)	87 (53.7%)	0.567
AKI stage at peak			0.835
Stage 1	12 (10.1%)	13 (8.0%)	
Stage 2	8 (6.7%)	11 (6.8%)	
Stage 3	99 (83.2%)	138 (85.2%)	
Severe comorbidities	11 (9.2%)	12 (7.4%)	0.579
Cost, US Dollars^a^	7944 (4248,16055)	5100 (2948,9396)	<0.001
In hospital mortality^b^	12 (10.6%)	7 (4.4%)	0.047
Treatment withdrawal^c^	23 (20.4%)	8 (5.0%)	<0.001
Renal-referral	80 (67.2%)	137 (84.6%)	0.001
Admission dept.			<0.001
ICU	60 (50.4%)	11 (6.8%)	
Renal	22 (18.5%)	88 (54.3%)	
Surgical	6 (5.0%)	27 (16.7%)	
Other	31 (26.1%)	36 (22.2%)	

*Abbreviations: CRRT* continuous renal replacement therapy, *RRT* renal replacement therapy, *AKI* acute kidney disease, *ICU* intensive care unit

^a^Missing value 36, ^b^Missing value 9, ^c^Missing value 9

## Discussion

There is an increasing incidence and prevalence of AKI globally [1, 7, 11] and severe AKI is associated with increased mortality up to greater than 50% [12–14]. In the absence of effective pharmacologic interventions for severe AKI, renal replacement therapy remains the main supportive management, and therefore is one of the critical aspects for improvement to achieve the goal of “ISN AKF 0 by 25”. However, up to now little is known about the need, availability, and maneuverability of RRT in the clinical practice in developing countries. Based on the nationwide survey of AKI in over 2 million adult hospitalizations, we were able to estimate the burden of RRT need and the real state of RRT performance in Mainland China [9].

Although there are some disagreements about the optimal timing for initiating RRT in AKI patients, there is no doubt that RRT should be performed in patients with life-threatening conditions including overt fluid imbalance, electrolyte abnormalities, acid-base disturbances, over accumulating metabolic toxins and uremic complications [15, 16]. According to these traditional RRT indications, we found that 11.8% of the AKI patients were in needs of RRT. From what we have reported in this nationwide survey as about 2.9 million AKI cases hospitalized in Mainland China during 2013 [9], it can be estimated that at least 342,200 AKI patients needed RRT treatment during their hospitalization in 2013. However, around 40% of these AKI patients needing RRT did not receive the treatment, and had significantly increased risk for mortality compared with those who were treated with RRT. Why had these patients been undertreated? As all the hospitals that were enrolled in the nationwide survey have facilities for IHD and PD, the significant situation of RRT under treatment cannot be attributed to the shortage of medical resources. We then tried to disclose potential affecting factors associated with patient and medical staff.

From multi-factorial analysis, we found that if the patients were older, in male gender, located in lower income areas, from local hospitals, or with malignancies and other severe comorbidities, they were more likely to be RRT under treated. This implies that socio-economic status might strongly affect the treatment choice for the individuals, and more consideration from the family would be made when their older and more severe relatives are in needs of RRT treatment. As male patients usually are the main support of their families, their sickness would directly affect the economic status of the family, and therefore the high medical cost for RRT treatment might be unaffordable. Besides, in some rural areas and minority regions, patients would prefer dying at home rather than in hospital, which could also contribute to the lack of RRT treatment in local hospitals. The high “treatment withdrawal” rate in these patients reinforces the above findings. Medical staff involved in patient care also played important roles in the current clinical status of RRT treatment selection. Patients who had oliguric AKI or stage 3 AKI, where the situation of renal failure easily arouses doctors’ attention, were of less risk for RRT under treatment. Moreover, those who were treated in renal department or received renal consultation had been significantly protected from RRT under treatment, which emphasizes the important impact of nephrologists on the treatment strategy determination in severe AKI patients.

Despite the significant under-treatment of RRT indicated patients in this survey, there was potential inappropriate prescription of CRRT treatment, which is an advanced but costy technique in treating critically ill patients. Since studies have shown that CRRT could benefit patients with sepsis, unstable hemodynamic, acute respiratory distress syndrome needing mechanical ventilation, MODS, and rhabdomyolysis [17–25], we then expanded the indications for CRRT according to these clinical conditions. We found that more than 1/3 of the patients were prescribed CRRT with unrecognized indication, of whom the survival was not improved while the cost was higher as compared to those treated with other RRT modalities. Therefore, CRRT could have been over prescribed in the clinical practice. What might be the reasons? Firstly, in the majority of the studied hospitals, CRRT facility is the only available RRT modality in the ICU departments and is performed by the ICU teams, therefore it is much more convenient to initiate CRRT than calling the nephrology team or transferring the patients to hemodialysis units. This can partially explain why most CRRT was prescribed in ICU departments. The fact that renal referral was associated with lower percentage of CRRT prescription in those patients who had no CRRT indications supports this possibility. Secondly, there could be a large variability in the understanding and decision of RRT modalities among different clinicians. The indications for CRRT have not been well established, and the implementation is more dependent on personal experience and preference in some circumstances. Besides, the very limited use of PD (only 1% in the current survey although with 100%availability in the study hospitals) makes CRRT the first choice in patients with unstable hemodynamic conditions. Investigations about the choice of RRT modalities in clinical practitioners would be helpful to disclose the real factors that influence their decisions, and enables improvements in the performance.

Therefore, the current study disclosed a noticeable under treatment rate in RRT indicated AKI patients, meanwhile for those who had been prescribed RRT, over treatment with CRRT could have also existed, which raises an important issue of rational use of medical resources. Although all of the hospitals in this study are capable of RRT, there still had been patients that could not afford RRT treatment, not to mention the numerous hospitals in rural areas that have no RRT resources. This is a common phenomenon in developing countries [26–28]. Therefore financial support from the government is in great need for helping the unaffordable conditions. On the other hand, proper utilization of RRT modalities, such as rational prescription of CRRT and more performance of PD, would help to increase the medical economics and save more lives of severe AKI with the limited medical resources. To achieve this goal, education and training for RRT practitioners is of most importance.

There are some limitations in this study. Firstly, as a retrospective study, we could not clearly clarify the causal relationship between treatments and outcomes. Secondly, classifications of clinical situation were made only on the basis of the medical records, thus the real clinical practice could not be fully recalled and biases may occur, such as underestimating of CRRT indicated patients. Finally, the inadequate measurement of SCr may underestimate the true need for RRT and renal recovery may not be able to compare with high missing value. However, this nation-wide survey is the largest and most representative of survey in AKI patients in China, from which we are able to reveal the current RRT situation in China and present some potential similarities in other developing countries.

## Conclusions

In summary, RRT has improved mortality in patients with severe AKI, however there have been a significant under treatment of RRT in China based on current retrospective study. CRRT might have been over-prescribed and the modalities of RRT need to be optimized to improve the medical economic efficiency in China. The situation that has been revealed in this study may represent a common status of RRT choice and performance in the real world of clinical practice in developing countries, calling for the awareness of appropriate utilization of RRT in severe AKI, a global, burdensome but treatable disease.

## Abbreviations

AKI: Acute kidney injury
CKD: Chronic kidney disease
CRRT: Continuous renal replacement therapy
CVD: Cardiovascular disease
DM: Diabetes mellitus
GDP: Gross domestic product
ICU: Intensive care unit
IHD: Intermittent hemodialysis
ISN: International Society of Nephrology
MODS: Multiple organ dysfunction syndrome
PD: Peritoneal dialysis
RRT: Renal replacement therapy
SCr: Serum creatinine
